# Transient Thermal Energy Harvesting at a Single Temperature Using Nonlinearity

**DOI:** 10.3390/e27040374

**Published:** 2025-03-31

**Authors:** Tamzeed B. Amin, James M. Mangum, Md R. Kabir, Syed M. Rahman, Ashaduzzaman, Pradeep Kumar, Luis L. Bonilla, Paul M. Thibado

**Affiliations:** 1 Department of Physics, University of Arkansas, Fayetteville, AR 72701, USA; tbamin@uark.edu (T.B.A.); jmmangum@uark.edu (J.M.M.); pradeepk@uark.edu (P.K.); 2 Materials Science and Engineering Program, University of Arkansas, Fayetteville, AR 72701, USA; kabir@uark.edu (M.R.K.); sr096@uark.edu (S.M.R.); a004@uark.edu (A.); 3 Nanoscience and Industrial Mathematics and Department of Mathematics, G. Millán Institute for Fluid Dynamics, Universidad Carlos III de Madrid, 28911 Leganés, Spain; bonilla@ing.uc3m.es

**Keywords:** transient dynamics, diode nonlinearity, storage capacitor, energy harvesting, stochastic simulations, Fokker–Planck, Ito–Langevin

## Abstract

The authors present an in-depth theoretical study of two nonlinear circuits capable of transient thermal energy harvesting at one temperature. The first circuit has a storage capacitor and diode connected in series. The second circuit has three storage capacitors, and two diodes arranged for full wave rectification. The authors solve both Ito–Langevin and Fokker–Planck equations for both circuits using a large parameter space including capacitance values and diode quality. Surprisingly, using diodes one can harvest thermal energy at a single temperature by charging capacitors. However, this is a transient phenomenon. In equilibrium, the capacitor charge is zero, and this solution alone satisfies the second law of thermodynamics. The authors found that higher quality diodes provide more stored charge and longer lifetimes. Harvesting thermal energy from the ambient environment using diode nonlinearity requires capacitors to be charged but then disconnected from the circuit before they have time to discharge.

## 1. Introduction

Recent advances in ultralow power circuit designs have dramatically reduced power consumption to nanowatts in active mode and picowatts in standby mode [1,2,3,4,5]. Low-power usage opens up the possibility of self-powering from ambient sources. This amount of power can easily be found from electromagnetic sources and even from mechanical vibrations in noisy environments. Even if the mechanical vibrations are stochastic in nature, they can be used as a source of power [6]. However, in a dark and quiet setting these sources are not available and only thermal energy at a single temperature is present.

Random electrical signals from thermal energy have a rich history going back to the 1920s when Johnson and Nyquist discovered electronic noise generated by charge carriers inside conductors in thermal equilibrium [7,8]. Once the diode was invented, it was natural to ask if diodes could rectify this noisy alternating current. This question was investigated by many notable scientists over the decades [9,10,11,12,13,14].

If two temperatures are present, then a diode can be used to harvest energy in the steady state [15,16]. Also, if the noise is non-Gaussian, other researchers discovered that one can then harvest energy from a single temperature [17,18,19,20,21]. As an example, our earlier experimental work found that the velocity distribution of a freestanding graphene membrane follows a heavy-tailed Lorentzian distribution [22,23].

More recently, our theoretical work discovered that one can harvest thermal energy directly from a single temperature using diode nonlinearity [24]. This is a transient phenomenon, and we showed that the detailed balance is temporarily broken to produce a non-zero direct current. Experimentally, just this year, thermal noise rectification has been achieved using a graphene-based ballistic diode utilizing the Tesla valve design [25].

Our previous study solved the Fokker–Planck (FP) equation for various circuits to prove energy harvesting at a single temperature. These are computationally intensive because one directly solves for the probability distribution for the charge on a capacitor in time [26,27]. As an example, it was not possible for us to solve circuits with more than two current junctions using FP. Ito–Langevin (IL) simulations, on the other hand, are capable of solving much more complex circuits as the output from each time step is a single value [28,29,30,31].

In this study, we numerically analyze two circuits using both the FP and IL methods. All circuit components are held at the same temperature. By using both approaches, we determine the largest possible IL time step that provides agreement with FP. The initial state of the storage capacitors is shorted to have zero charge, variance, and entropy. From a physical point of view, we imagine connecting a wire with zero resistance across the capacitor, forcing the charge distribution to be a delta function centered at zero charge, and then we remove the wire to start the dynamics (i.e., the Nyquist voltage variance is zero for zero resistance). The temperature is always the same. We then track the charge, variance, and entropy in time using a large range of parameter values.

## 2. Results and Discussion

### 2.1. Single Loop Circuit

**Figure 1:**
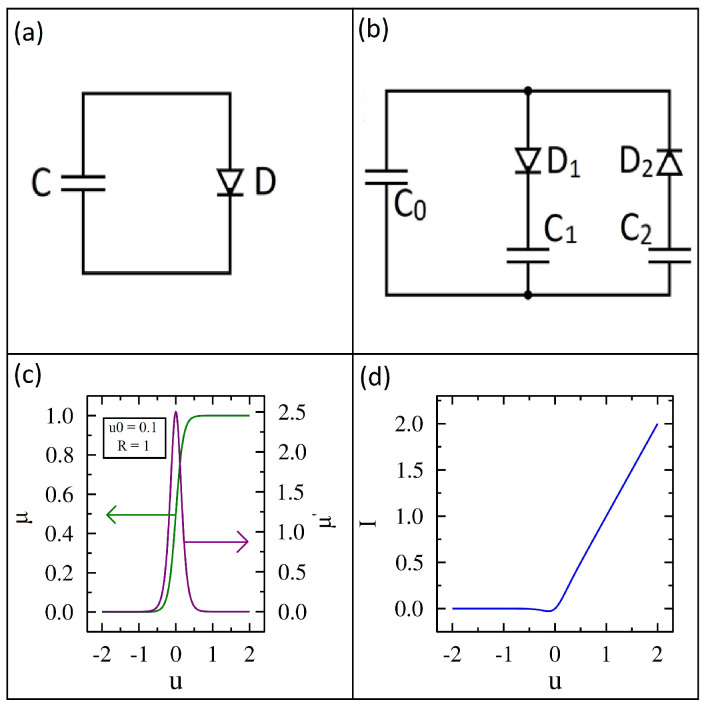
Two circuits analyzed in this study and diode properties shown with R=1 and $u_{0}=0.1$. (**a**) Diode−capacitor circuit. (**b**) Two diode and three capacitor circuit. (**c**) Diode conductance (green curve) and its derivative (purple curve) shown versus diode voltage. (**d**) Diode current−voltage characteristics.

The first circuit we analyze consists of a capacitor in series with a diode, as shown in Figure 1a. The diode orientation allows positive charge carriers to flow clockwise with minimal resistance. The energy of this circuit is given by

(1) $$H\left(q\right)=\frac{q^{2}}{2C},\;$$

where H is the Hamiltonian, *q* is the charge on the capacitor, and *C* is its capacitance.

For the diode, we model its directional-dependent conductance, μ, using a sigmoid function given by

(2) $$\mu \left(u\right)=\frac{1}{R}\frac{1}{1+e^{-u/u_{0}}},$$

where *R* is its resistance at high-forward bias, *u* is the voltage drop across the diode, and $u_{0}$ controls its quality. Notice that μ depends on *q* through *u*.

The diode’s conductance versus bias voltage is plotted for R=1 and $u_{0}=0.1$ in Figure 1c. The conductance is near zero for negative bias voltages and rises quickly to unity for positive bias voltages. As the diode parameter $u_{0}$ approaches zero, the diode becomes a step function or an ideal switch. As $u_{0}$ approaches infinity, the diode has a constant conductance of 1/2 for all bias voltages and the diode becomes a linear resistor.

The derivative of the conductance is also shown in Figure 1c. Its value is zero except close to zero bias, where its value is 2.5. We will see later that this non-zero derivative value at zero bias volts drives the initial current that charges the storage capacitor. The current-voltage (I−u) characteristics of the diode are shown in Figure 1d. The I−u curve is similar to an ideal diode in series with a resistor, which at high forward bias is more realistic than the Shockley diode equation [32].

#### 2.1.1. Differential Equations

The IL equation for the circuit shown in Figure 1a in terms of the conductance and Hamiltonian is given by

(3) $$dq=\left(k_{B}T\frac{\partial \mu }{\partial q}-\mu \frac{\partial H}{\partial q}\right)dt+\sqrt{2\mu k_{B}T}\;d\zeta_{q}\left(t\right),\;$$

where $k_{B}$ is the Boltzmann constant, *T* is the absolute temperature, *t* is time, and $d\zeta_{q}$ is delta function correlated Gaussian noise with mean zero and variance dt. Notice the first term on the right-hand side is $k_{B}T$ times the derivative of μ. As alluded to earlier, this term produces the initial direct current at zero bias and places a net charge on the capacitor.

The equivalent FP equation for this circuit is given by

(4) $$\frac{\partial \rho }{\partial t}=\frac{\partial }{\partial q}\left(\mu \rho \frac{\partial H}{\partial q}\right)+k_{B}T\frac{\partial }{\partial q}\left(\mu \frac{\partial \rho }{\partial q}\right),\;$$

where ρ(q,t) is the probability distribution for the charge on the capacitor in time. The derivation of our IL and FP equations can be found here [23,24].

#### 2.1.2. Simulations

We performed numerical simulations of Equations (3) and (4). We use the first-order Milstein numerical scheme for IL, and for FP we use an explicit forward in time and centered in space scheme on a square grid in *q* with Δq=0.01 and $\Delta t=10^{-5}$ [33]. The averages presented from Equation (3) are found after performing 3000 realizations. For this first circuit, we present most of our results in terms of dimensionless variables, which helps us present the large range of parameter values used in the study.

**Figure 2:**
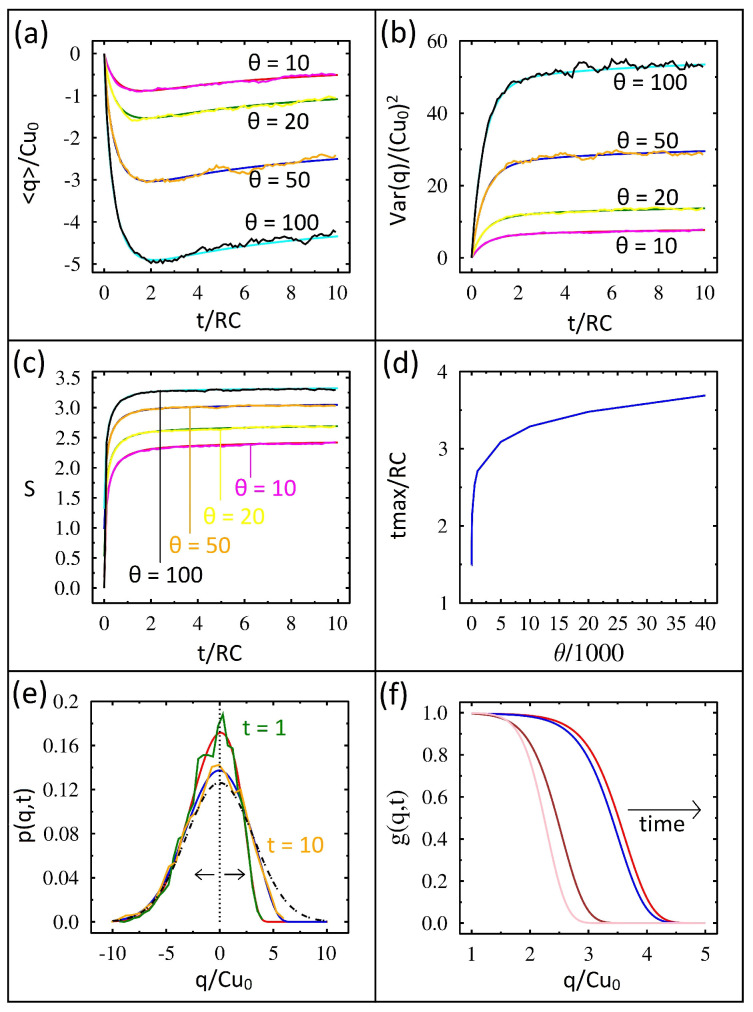
Numerical solutions of FP (smooth lines) and IL (jagged lines) equations for the diode−capacitor circuit shown in terms of dimensionless variables. (**a**) Capacitor charge vs. time for four temperatures. (**b**) Variance vs. time for four temperatures. (**c**) Shannon entropy vs. time for four temperatures. (**d**) Time to reach maximum charge vs. temperature. (**e**) Probability distribution vs. charge at two simulation times. At time t=1, FP (red curve) and IL (green curve). At time t=10 FP, (blue curve) and IL (yellow curve). The vertical dashed line at zero charge represents the initial conditions. The two arrows indicate the spreading direction in time. The equilibrium solution is shown as a dotted−dashed line. (**f**) Time evolution of the probability distribution divided by the equilibrium distribution vs. charge at four simulation times. The left two curves are at times 1 and 2, while the right two curves are at times 5 and 6.

The charge on the capacitor, $q/Cu_{0}$, in time, t/RC, is shown in Figure 2a. The four plots represent different temperatures, $\theta =k_{B}T/Cu_{0}^{2}$. The IL solutions are the jagged lines, while the FP solutions are smooth. The average charge values for IL are in excellent agreement with those found using FP. However, this agreement only occurs at sufficiently short IL time steps. The simulations require times $dt=10^{-6}$ for this agreement. When dt is larger, the capacitor may remain charged for all time (i.e., revealing an integration error). For all of the temperatures studied, the capacitor initially charges, reaches a maximum charge, and then begins to discharge. Notice that higher temperatures have a higher rate of initial charging. Furthermore, higher temperatures reach a higher overall maximum negative charge.

It is easiest to understand the capacitor charging results if we first consider the equilibrium solution. As mentioned previously, the capacitor begins with zero charge and zero variance, and in equilibrium it will also have zero charge but its variance will be $k_{B}TC$. If the diode was a resistor, then the charge would remain zero for all time, while the variance would grow from zero to $k_{B}TC$ with a time constant of RC. However, the directional conductance of the diode changes the dynamics. With a small forward-bias resistance, the variance quickly reaches equilibrium for negative charges. With a large reverse-bias resistance, the variance requires an extremely long time to reach equilibrium for positive charges. Consequently, the capacitor quickly builds up a negative charge and then slowly discharges. Furthermore, higher temperatures will have higher rates of initial charging and as a result reach a higher maximum charge.

We can quantify, to some degree, the slow capacitor discharge if we scale the charge in the FP equation with $Cu_{0}$. Then, the eigenvalue λ, obtained by separating variables, scales as $\lambda \times {\left(Cu_{0}\right)}^{2}$, which tends to zero with $u_{0}$. This shows that the time scale (proportional to 1/eigenvalue) becomes longer as $u_{0}$ tends to zero. This scaling is a consequence of the two derivatives of the probability density that appear in the FP equation [24].

The variance (dimensionless) in time for the same four temperatures is shown in Figure 2b. Excellent agreement between IL and FP is found. In all cases, the variance starts at zero and rapidly rises before plateauing. As expected from the equilibrium solution mentioned earlier, systems held at a higher temperature have a higher variance.

The entropy in time at the different temperatures is shown in Figure 2c. Again, we have excellent agreement between IL and FP. Entropy is calculated using the Shannon entropy, S=−〈lnρ〉 [30]. One can understand the entropy results by again looking at the equilibrium solution, where $S_{eq}=\ln(2\pi e$ variance)/2. Given that the equilibrium entropy is simply the logarithm of the equilibrium variance, the entropy changes mirror those of the variance.

The time required for the capacitor to reach its maximum charge, $t_{max}$, as a function of temperature is shown in Figure 2d. The time plotted here is again dimensionless and has been divided by the characteristic charging time, RC; surprisingly the scaled time $t_{max}/RC$ significantly increases with temperature. To understand this result, we must again consider the properties of the diode. In particular, the derivative of the conductance is multiplied by the temperature in Equation (3) as mentioned earlier. This factor drives the initial charging higher, and our $t_{max}$ data highlight the significant impact of diode nonlinearity.

The time evolution of the probability distribution is shown in Figure 2e. Initially, the probability distribution is the vertical dashed line shown at zero charge. This distribution has zero charge and zero variance. At equilibrium, the probability distribution is the dash-dotted Gaussian curve. At simulation time, t=1, the FP probability distribution and the IL probability distribution are the highest two curves and are in excellent agreement. Notice the negative charge side of the probability distributions has already reached equilibrium. The probability distribution at time t=10 is also shown, and it has moved closer to the equilibrium distribution for positive charges but is still far away.

We can understand these results by again considering the diode’s asymmetric conductance. The forward bias has a very low resistance compared to the reverse bias; therefore, the negative charges reach equilibrium quickly, while the positive charges require an extremely long time to reach equilibrium.

We plot the function $g\left(q,t\right)=\rho \left(q,t\right)/\rho_{eq}$ in Figure 2f at four different times (the solution found from the advection–diffusion equation). This function nicely captures the flow in time to equilibrium. Since the system has already reached equilibrium for negative charges, this function is one for negative charge values. However, for positive charges the probability is far below equilibrium and thus g(q,t) is close to zero. The right edge or front of this function is moving to the right in time. For example, the left-most pair of lines shows the function at times t=1 and t=2. Notice how far the function has moved to the right in one time step. The right-most pair of lines shows the function at times t=5 and t=6. Notice how much further the function has progressed to the right. However, more importantly, notice the lack of comparable progress for the additional time step. What we found is that the approach to equilibrium is slowing down in time, and this plot captures that [24].

### 2.2. Two Loop Circuit

The second circuit we studied has two current junctions, three capacitors, and two diodes, as shown in Figure 1b. The conductance function for the diodes is identical to the earlier section. For this circuit, from the perspective of capacitor $C_{0}$ the diodes are wired in opposition for full-wave rectification. Capacitor $C_{0}$ experiences forward bias (low resistance) for current flowing from above and below, while the other two capacitors see forward bias for current flowing in only one direction. This property will play an important role in understanding the performance of the circuit. The energy of this circuit is given by

(5) $$H\left(q_{1},q_{2}\right)=\frac{{\left(q_{1}+q_{2}\right)}^{2}}{2C_{0}}+\frac{q_{1}^{2}}{2C_{1}}+\frac{q_{2}^{2}}{2C_{2}},\;$$

where $q_{1}$ and $q_{2}$ are the charges on $C_{1}$ and $C_{2}$, respectively. Due to the junction rule and the initial charge being set to zero, the charge on $C_{0}$ is $q_{1}+q_{2}$. For all the results presented here, we set $C_{1}=C_{2}$. The simulations give identical results for both capacitors but opposite in sign; therefore, we present only those for $C_{1}$.

#### 2.2.1. Differential Equations

The IL equations for the charge on capacitors $C_{1}$ and $C_{2}$ are given by

(6) $$dq_{1}=\left(k_{B}T\frac{\partial \mu_{1}}{\partial q_{1}}-\mu_{1}\frac{\partial H}{\partial q_{1}}\right)dt+\sqrt{2\mu_{2}k_{B}T}\;d\zeta_{1}\left(t\right)\;$$

(7) $$dq_{2}=\left(k_{B}T\frac{\partial \mu_{2}}{\partial q_{2}}-\mu_{2}\frac{\partial H}{\partial q_{2}}\right)dt+\sqrt{2\mu_{2}k_{B}T}\;d\zeta_{2}\left(t\right),$$

where $\zeta_{1}\left(t\right)$ and $\zeta_{2}\left(t\right)$ are zero mean and independent white noise.

The equivalent FP equations for this circuit are given by

(8) $$\frac{\partial \rho }{\partial t}=\sum_{i=1}^{2}\left[\frac{\partial }{\partial q_{i}}\left(\mu_{i}\rho \frac{\partial H}{\partial q_{i}}\right)+k_{B}T\frac{\partial }{\partial q_{i}}\left(\mu_{i}\frac{\partial \rho }{\partial q_{i}}\right)\right].$$

#### 2.2.2. Simulations—Charge Variance

For this more complicated circuit, we begin by presenting the results for the variance of the charge on capacitor $C_{1}$, instead of its average charge. We set the initial variance to zero, and we calculated the final variance using Equation (5). Knowing the extrema helps explain the dynamics. We performed numerical simulations of Equations (6)–(8) and averaged 3000 realizations for IL. We generally set $k_{B}T=1$ and R=1 for these simulations.

**Figure 3:**
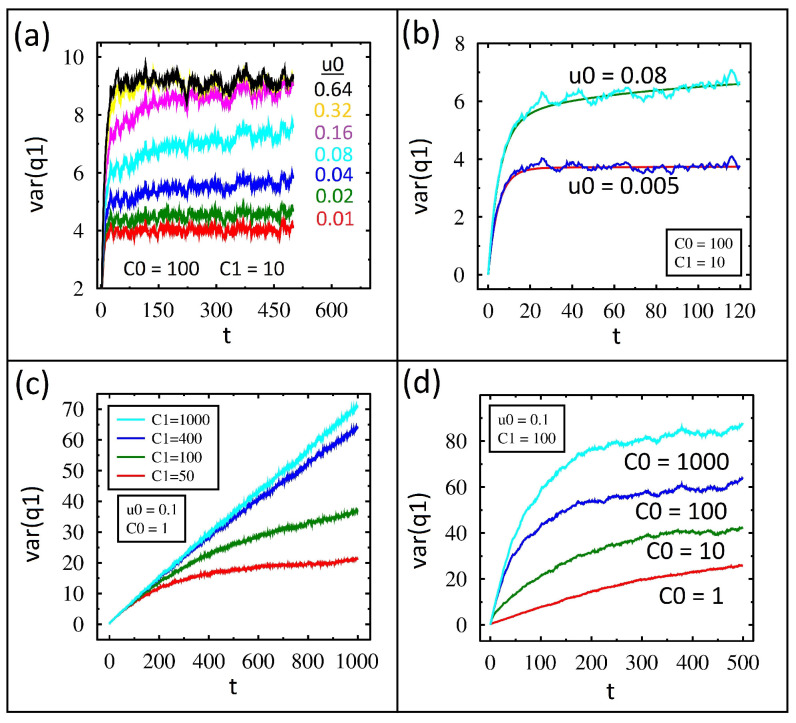
Charge variance on $C_{1}$ using $k_{B}T=1$ and R=1. (**a**) Time dependence of charge variance from IL for different values of $u_{0}$ and fixed values of $C_{1}=C_{2}=10$ and $C_{0}=100$. (**b**) Comparison of FP (smooth lines) and IL (jagged lines) for fixed values of $C_{1}=C_{2}=10$ and $C_{0}=100$ while $u_{0}$ changes from 0.005 to 0.08. (**c**) Variance for different values of $C_{1}$ with fixed values of $C_{0}=1$ and $u_{0}=0.1$. (**d**) Variance for different values of $C_{0}$ with fixed values of $u_{0}=0.1$ and $C_{1}=100$.

The charge variance in time from IL for various values of the diode parameter, $u_{0}$, is shown in Figure 3a. The value of $u_{0}$ is shown adjacent to its particular data plot, and we set $C_{0}=100$ and $C_{1}=10$ for all of these runs. For each data set, the variance begins at zero, rises for a short period of time, and then plateaus. Lower values of $u_{0}$ show a lower value of variance at the end of the simulation.

To understand these results, first notice the equilibrium variance is nine, as shown in the second row in column three of Table 1. The top three variance plots have reached nine by the end of the simulation, and these all have high values of $u_{0}$, which means the diodes are closer to a resistor than a switch. The plots with smaller values of $u_{0}$ will clearly require a lot more time to reach equilibrium. This is because the diode primarily conducts in one direction, and the high reverse bias resistance yields a very long time constant.

The variance in time for two $u_{0}$ values for both IL and FP is shown in Figure 3b. Excellent agreement between the two methods is found. Notice how the variance for the smaller $u_{0}$ value is extremely flat for most of the simulation even though it too must reach nine at some point in time.

The charge variance in time from IL for various values of capacitance $C_{1}$ is shown in Figure 3c. Lower values of $C_{1}$ rise and level off during the simulation time, while higher values have nearly linear growth in time.

In order to understand these results, it is helpful to know the equilibrium variance. The value was calculated using Equation (5) and listed in column three of Table 1 in the next four rows. Near the end of the variance plots, for small capacitance values, notice the equilibrium value is nearly reached, and this is why the curves start to level off. For large capacitance values, the plots are not leveling off. In other words, the larger capacitance values take longer to reach equilibrium, but this is consistent with RC being the time constant.

The variance over time from IL for various $C_{0}$ values is shown in Figure 3d. After close inspection, the dynamics of these are opposite to those shown in Figure 3c. For example, when $C_{0}=1000$ the variance rises but then plateaus, but when $C_{0}=1$ the variance grows almost linearly throughout the simulation time.

To understand these results, we need more information than just the capacitance and the equilibrium variance increasing in value. Notice, the equilibrium variance is shown in Table 1. When $C_{0}=1000$, the equilibrium variance is 92, and by the end of the simulation the value is already close to 90. So, it has almost reached equilibrium already. At the other extreme, when $C_{0}=1$ the equilibrium variance is 50, but the simulation has only reached half of this value. So, even though $C_{0}$ is decreasing, the time constant for the circuit is actually increasing.

The reason for this can be understood by considering the resistance of the diodes in the circuit. When $C_{1}$ is large compared to $C_{0}$, then the lowest electrical reactance path for the current is circulating between $C_{1}$ and $C_{2}$. For this current path, the diodes are in series with a low resistance for the counterclockwise current and a high resistance for the clockwise current. However, when $C_{1}$ is small compared to $C_{0}$, then the lowest reactance path for the current is circulating through $C_{0}$. There are two possible paths for this current, either through $D_{1}$ or $D_{2}$. Since the diodes are wired in opposition, both current paths will have low resistance to $C_{0}$. This significantly lowers the overall resistance and thus the time constant, as the reverse bias of the diodes is not needed to reach equilibrium.

To further support this concept, we present the equivalent capacitance for current paths that both include and exclude $C_{0}$, as shown in the last two columns of Table 1. The largest equivalent capacitance between the two columns then determines the primary path of the current flow and hence the resistance. When $C_{0}=1000$, the $C_{0}$ capacitance path is 91 versus 50 for $C_{1}$, so the $C_{0}$ path wins. However, when $C_{1}=1000$ the $C_{1}$ capacitance path is 500 versus 1. We will revisit the effective time constant when we present the average charge formed on the capacitors later on.

#### 2.2.3. Simulations—Entropy

**Figure 4:**
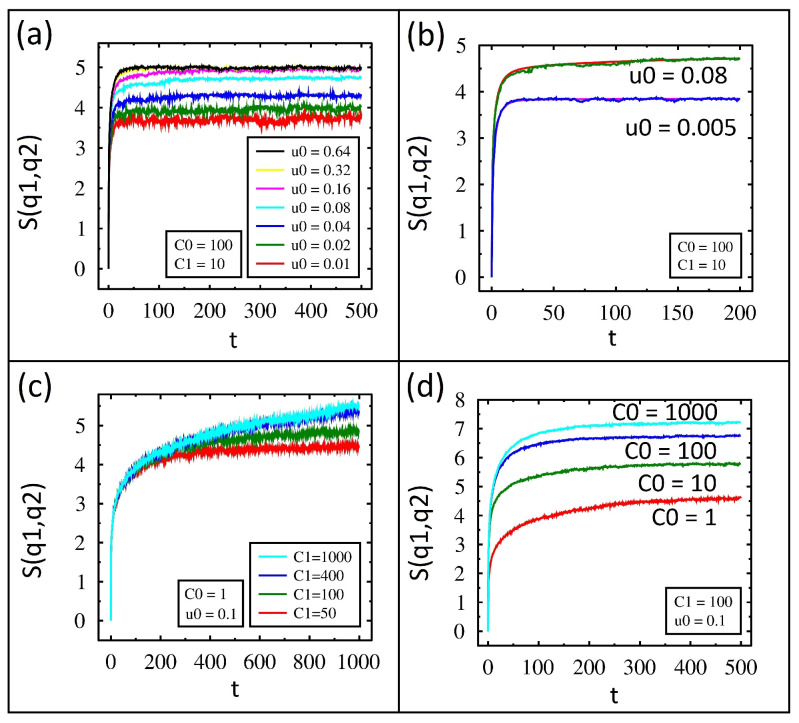
Numerical solutions for entropy using $k_{B}T=1$ and R=1. (**a**) Time dependence of entropy from IL for different values of $u_{0}$ and fixed values for $C_{1}=C_{2}=10$ and $C_{0}=100$. (**b**) Comparison of FP (smooth lines) and IL (jagged lines) for fixed values of $C_{1}=C_{2}=10$ and $C_{0}=100$ while $u_{0}$ changes from 0.005 to 0.08. (**c**) Entropy for different values of $C_{1}$ and fixed values of $C_{0}=1$ and $u_{0}=0.1$. (**d**) Entropy for different values of $C_{0}$ and fixed values of $u_{0}=0.1$ and $C_{1}=C_{2}=100$.

The charge entropy in time from IL for various values of the diode parameter, $u_{0}$, is shown in Figure 4a. The value of $u_{0}$ is shown in the same order as the data plots. For each data set, the entropy begins at zero, rises very quickly for a short period of time, and then plateaus. Also, notice that the lower values of $u_{0}$ show a lower value of entropy at the end of the simulation. As expected, these results track the variance results presented and discussed earlier.

The entropy in time for $u_{0}=0.08$ and $u_{0}=0.005$ and is shown for both IL and FP in Figure 4b. Excellent agreement between the two methods is found. The charge entropy in time from IL for various values of capacitance $C_{1}$ is shown in Figure 4c. Lower values of $C_{1}$ rise but then level off during the simulation time. Higher values of $C_{1}$ show nearly linear growth in entropy. These results follow the variance as the entropy is simply related. The entropy over time from IL for various $C_{0}$ values is shown in Figure 4d. After close inspection, the dynamics of these results are opposite to those of Figure 4c. When $C_{0}=1000$ the entropy rises but then plateaus. When $C_{0}=1$, the entropy grows throughout the simulation time. As expected, these results also follow the same trend as the variance.

#### 2.2.4. Simulations—Average Charge

**Figure 5:**
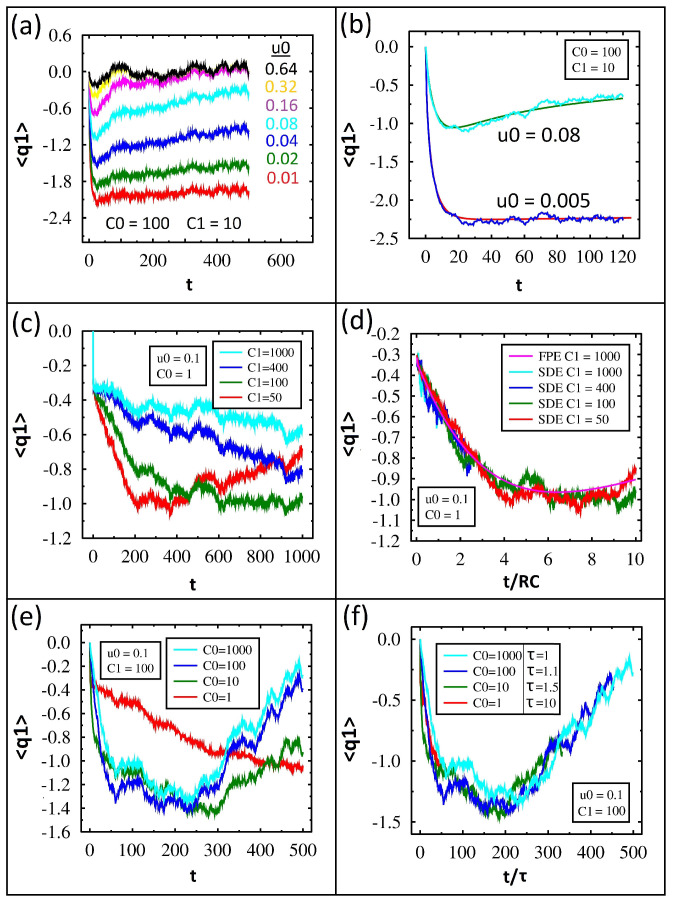
Numerical solution for the average charge on $C_{1}$ with $k_{B}T$ = 1 and *R* = 1. (**a**) Time-dependent average charge from IL when varying $u_{0}$ with fixed $C_{1}=C_{2}=10$ and $C_{0}=100$. (**b**) FP (smooth lines) and IL (jagged lines) solutions for average charge with fixed $C_{1}=C_{2}=10$, $C_{0}=100$ and varying $u_{0}$ from 0.005 to 0.08. (**c**) Average charge when varying $C_{1}$ with fixed $C_{0}=1$ and $u_{0}=0.1$. (**d**) Average charge comparison of FP and IL for varying $C_{1}=C_{2}$ vs. the scaled time, t/RC. (**e**) Average charge when varying $C_{0}$ with fixed $u_{0}=0.1$ and $C_{1}=100$. (**f**) Average charge when varying $C_{0}$ vs. the scaled time, t/τ, with each value of τ listed in the legend.

The charge in time on $C_{1}$ from IL for various values of the diode parameter, $u_{0}$, is shown in Figure 5a. The value of $u_{0}$ is shown adjacent to its particular data plot. For each data set, the charge begins at zero, decreases for a short period of time, and then plateaus. Lower values of $u_{0}$ show a higher negative charge on the capacitor at the end of the simulation.

We can understand these results from our earlier discussion. Only the top three charge plots have reached zero, which is equilibrium, by the end of the simulation. This is consistent with the variance reaching equilibrium, as discussed earlier, and is due to the diodes being similar to resistors. All the other plots with smaller values of $u_{0}$ will require more time to reach equilibrium, and as a result they are still charged. As the diode parameter approaches zero, the diode becomes an ideal switch, so it conducts current primarily in one direction. In other words, the system can charge quickly, but it discharges very slowly.

The charge in time for $u_{0}=0.08$ and $u_{0}=0.005$ is shown for both IL and FP in Figure 5b. Excellent agreement between the two methods is found. Notice how the charge for the smaller $u_{0}$ value is extremely large and flat for most of the simulation even though it must reach zero at some point in time.

The charge in time from IL for various values of capacitance $C_{1}$ is shown in Figure 5c. Lower values of $C_{1}$ show the charge decrease, level off, and begin discharging during the simulation time, while higher values only charge.

As with the variance discussion earlier, we found that the time constant for the circuit follows RC and thus increases with increasing capacitance. In order to show the time constant is the only property changing for the various capacitor values, we replot the charge versus t/RC for both IL and FP in Figure 5d. Notice how all the data plots collapse on each other and also have excellent agreement with the FP solution.

The charge in time from IL for various $C_{0}$ values is shown in Figure 5e. After close inspection, the dynamics of these are opposite those shown in Figure 5c. When $C_{0}=1000$, the capacitor charges, levels off, and discharges. When $C_{0}=1$, the capacitor only charges throughout the simulation time.

As with the variance discussion earlier, we found that the resistance of the circuit drops considerably as the value of $C_{0}$ increases. In order to quantify this, we scaled the capacitor charge for various values of $C_{0}$ with a time constant, τ, as shown in Figure 5f. The required value for τ to collapse the data is written in the legend adjacent to the value of $C_{0}$. Notice larger $C_{0}$ values require smaller τ values, which is consistent with the resistance significantly decreasing.

## 3. Conclusions

In summary, the authors studied two circuits by solving both Ito–Langevin (IL) and Fokker–Planck (FP) equations. The first circuit has a single diode in series with a capacitor. The charge, variance, and entropy dynamics were presented for various temperatures. In all cases, the capacitor charged quickly and discharged slowly, with the maximum charge reached increasing with temperature and diode quality.

The second circuit consists of two diodes, three capacitors, and two current junctions. The diodes are oriented in opposite directions for full-wave rectification. Diode quality and capacitance values were varied over a large range. The capacitors again quickly charged and slowly discharged, and the maximum charge reached was higher for higher quality diodes. The variance and entropy were also presented. The authors found that the maximum charge achieved occurs when the capacitance of $C_{0}$ is much smaller than the capacitance of the storage capacitors $C_{1}$ and $C_{2}$. The charges stored are equal in magnitude but opposite in sign.

Solutions using FP require significant computing power compared to IL. As a result, FP may only be used for relatively simple circuits. The authors found excellent agreement between IL and FP, but only when the time step for IL is sufficiently small. Nevertheless, this result opens up the possibility of using IL with confidence for much more complex circuits in the future.

Surprisingly, using diodes one can harvest thermal energy at a single temperature by charging capacitors. However, this is a transient phenomenon. In equilibrium, the capacitor charge is zero, and this solution alone satisfies the second law of thermodynamics. The authors found that higher quality diodes provide more stored charge and longer lifetimes. Harvesting thermal energy from the ambient environment using diode nonlinearity requires capacitors be charged but then disconnected from the circuit before they have time to discharge.

## Figures and Tables

**Table 1:** Calculated equilibrium charge variance values and equivalent capacitance values for the circuit shown in Figure 1b for various combinations of $C_{0}$ and $C_{1}$ used in this study.

$C_{0}$	$C_{1}=C_{2}$	$\mathrm{Var}(q_{1})$	$\left(C_{0}\times C_{1}\right)/\left(C_{0}+C_{1}\right)$	$\left(C_{1}\times C_{2}\right)/\left(C_{1}+C_{2}\right)$
100	10	9	9	5
1	50	25	1	25
1	100	50	1	50
1	400	190	1	200
1	1000	490	1	500
1	100	50	1	50
10	100	52	9	50
100	100	66	50	50
1000	100	92	91	50

## Data Availability

The original contributions presented in this study are included in the article. Further inquiries can be directed to the corresponding author.

## Abbreviations

The following abbreviations are used in this manuscript: FP Fokker–Planck, IL Ito–Langevin, H Hamiltonian, *q* charge, *C* capacitance, *R* resistance, *u* voltage, $u_{0}$ diode parameter, *μ* conductance, *k_B_* Boltzmann constant, *T* absolute temperature, *t* time, $d\zeta_{q}$ delta function correlated Gaussian noise, *ρ*(*q*, *t*) probability distribution, *ρ_eq_* equilibrium probability distribution, *τ* time constant, *λ* eigenvalue, *t_max_* time at maximum charge, and *S* Shannon entropy.

## References

[1] Banerjee A., Farhoudi N., Ghosh C., Mastrangelo C.H., Kim H., Broadbent S.J., Looper R. Picowatt gas sensing and resistance switching in gunneling nano-gap electrodes. Proceedings of the 2016 IEEE SENSORS.

[2] Hanson S., Seok M., Lin Y.-S., Foo Z., Kim D., Lee Y., Liu N., Sylvester D., Blaauw D. (2009). A low-voltage processor for sensing applications with picowatt standby mode. IEEE J. Solid-State Circuits.

[3] Lee Y., Seok M., Sylvester S., Blaauw D. (2013). Achieving ultralow standby power with an efficient SCCMOS bias generator. IEEE Trans. Circuits Syst. II Express Briefs.

[4] Basu J., Ali K., Lin L., Alito M. (2022). Picowatt-pwer analog gain stages in super-cutoff region with purely-harvested demonstration. IEEE-Solid-State Circuits Lett..

[5] Gupta N., Makosiej A., Anghel C., Amara A., Vladimirescu A. (2016). CMOS sensor nodes with sub-picowatt TFET memory. IEEE Sens. J..

[6] Costanzo L., Schiavo A.L., Sarracino A., Vitelli M. (2024). Stochastic thermodynamics of an electromagnetic energy harvester. Entropy.

[7] Johnson J.B. (1928). Thermal agitation of electricity in conductors. Phys. Rev..

[8] Nyquist H. (1928). Thermal agitation of electric charge in conductors. Phys. Rev..

[9] Brillouin L. (1950). Can the rectifier become a thermodynamical demon?. Phys. Rev..

[10] Gunn J.B. (1968). Thermodynamics of nonlinearity and noise in diodes. J. Appl. Phys..

[11] Gunn J.B., Staples J.L. (1969). Spontaneous reverse current due to the Brillouin EMF in a diode. Appl. Phys. Lett..

[12] van Kampen N.G. (1957). Derivation of the phenomenological equations from the master equation: I. Even variables only. Physica.

[13] van Kampen N.G. (1960). Non-linear thermal fluctuations in a diode. Physica.

[14] Feynman R.P., Leighton R.B., Sands M. (1966). The Feynman Lectures on Physics.

[15] Sokolov I.M. (1998). On the energetics of a nonlinear system rectifying thermal fluctuations. Europhys. Lett..

[16] Sokolov I.M. (1999). Reversible fluctuation rectifier. Phys. Rev. E.

[17] Magnasco M.O. (1993). Forced Thermal Ratchets. Phys. Rev. Lett..

[18] Doering C.R., Horsthemke W., Riordan J. (1994). Nonequilibrium fluctuation-induced transport. Phys. Rev. Lett..

[19] Filliger R., Reimann P. (2007). Brownian gyrator: A minimal heat engine on the nanoscale. Phys. Rev. Lett..

[20] Chiang K.-H., Lee C.-L., Lai P.-Y., Chen Y.-F. (2017). Electrical autonomous Brownian gyrator. Phys. Rev. E.

[21] Gonzalez J.P., Neu J.C., Teitsworth S.W. (2019). Experimental metrics for detection of detailed balance violation. Phys. Rev. E.

[22] Ackerman M.L., Kumar P., Neek-Amal M., Thibado P.M., Peeters F.M., Singh S. (2016). Anomalous Dynamical Behavior of Freestanding Graphene Membranes. Phys. Rev. Lett..

[23] Thibado P.M., Kumar P., Singh S., Ruiz-Garcia M., Lasanta A., Bonilla L.L. (2020). Fluctuation-induced current from freestanding graphene. Phys. Rev. E.

[24] Thibado P.M., Neu J.C., Kumar P., Singh S., Bonilla L.L. (2023). Charging capacitors from thermal fluctuations using diodes. Phys. Rev. E.

[25] Nguyen V.H., Kim M., Suleman M., Nasir N., Park H.M., Lee S., Elahi E., Noh H., Kumar S., Seo Y. (2025). Thermal noise rectification with graphene. Nano Energy.

[26] Fokker A.D. (1914). Die mittlere Energie rotierender Elektrischer dipole im Strahlungsfeld. Ann. Phys..

[27] Planck V.M. (1917). Über Einen Satz der Statistischen Dynamik und Seine Erweiterung in der Quantentheorie.

[28] Hjelmfelt A., Ross J. (1992). Thermodynamics and stochastic theory of electrical circuits. Phys. Rev. A.

[29] Sekimoto K. (2010). Stochastic Energetics.

[30] Seifert U. (2012). Stochastic thermodynamics, fluctuation theorems and molecular machines. Rep. Prog. Phys..

[31] Durbin J., Mangum J.M., Gikunda M.N., Harerimana F., Amin T., Kumar P., Bonilla L.L., Thibado P.M. (2023). Freestanding graphene heat engine analyzed using stochastic thermodynamics. AIP Adv..

[32] Sze S.M. (1981). Physics of Semiconductor Devices.

[33] Gardiner C.W. (2010). Stochastic Methods: A Handbook for the Natural and Social Sciences.

